# Questionnaire-Based Assessment of Obstructive Sleep Apnea Risk During Pregnancy: Associations with Metabolic Parameters and Family History of Diabetes

**DOI:** 10.3390/healthcare14162535

**Published:** 2026-08-13

**Authors:** Hüseyin Karakaya, Gökhan Doğukan Akarsu, Rukiye Höbek Akarsu

**Affiliations:** 1 Department of Obstetrics and Gynecology, Faculty of Medicine, Yozgat Bozok University, 66100 Yozgat, Türkiye; drhusyn@gmail.com; 2 Department of Pharmacy Services, Vocational School of Health Services, Yozgat Bozok University, 66100 Yozgat, Türkiye; 3 Department of Nursing, Faculty of Health Sciences, Yozgat Bozok University, 66100 Yozgat, Türkiye; rukiye-hobek@hotmail.com

**Keywords:** good health and well-being (SDG 3), STOP-BANG, Berlin Questionnaire, obstructive sleep apnea, pregnancy, metabolic parameters, family history of diabetes

## Abstract

**Objective:** Obstructive sleep apnea (OSA) during pregnancy is associated with adverse maternal and fetal outcomes, yet commonly used screening questionnaires may perform differently because of pregnancy-specific physiological changes. This study compared questionnaire-based OSA risk classifications obtained using the Berlin Questionnaire and STOP-BANG and examined their associations with metabolic parameters and diabetes-related history. **Methods:** This cross-sectional study included 210 pregnant women attending a tertiary obstetrics clinic. Berlin Questionnaire high risk was defined as positivity in at least two categories. STOP-BANG scores of 0–2, 3–4, and 5–8 indicated low, moderate, and high risk, respectively. Sociodemographic, obstetric, anthropometric, and biochemical variables were analyzed using multivariable logistic regression. **Results:** The Berlin Questionnaire classified 34 participants (16.2%) as high risk. STOP-BANG classified 183 (87.1%) as low risk, 5 (2.4%) as moderate risk, and 22 (10.5%) as high risk. Higher current weight was associated with increased questionnaire-based OSA risk according to both tools. In the Berlin model, HbA1c, BMI, and family history of diabetes were independently associated with high questionnaire-based OSA risk. No independent associations were identified in the STOP-BANG model. Although the Berlin model had 83.3% overall accuracy, it correctly classified only 2.9% of high-risk participants. **Conclusions:** The questionnaires identified different patterns of OSA risk. Findings were instrument-specific and do not indicate confirmed OSA. Given the absence of polysomnography, modest model performance, and limited high-risk classification, the results should be interpreted cautiously.

## 1. Introduction

Pregnancy is associated with profound physiological, hormonal, and anatomical changes that may adversely affect sleep quality and respiratory function. Progressive weight gain, upper airway mucosal edema, and reduced functional residual capacity increase susceptibility to sleep-disordered breathing throughout gestation. Although sleep disturbances become more common as pregnancy progresses, symptoms such as excessive daytime sleepiness and obstructive sleep apnea (OSA) may also occur during early pregnancy [1,2,3].

OSA is increasingly recognized as an important maternal health concern because of its association with adverse pregnancy outcomes, including gestational hypertension, preeclampsia, gestational diabetes mellitus (GDM), fetal growth restriction, preterm birth, and perinatal depression [4,5,6,7]. Recent evidence suggests that OSA affects approximately 15–20% of pregnant women, although reported prevalence varies according to the characteristics of the study population and the screening or diagnostic methods used [6].

Gestational diabetes mellitus is one of the most common metabolic complications of pregnancy and is associated with increased maternal and neonatal morbidity [8]. Previous studies have suggested a bidirectional relationship between OSA and glucose metabolism. Intermittent hypoxia and sleep fragmentation associated with OSA may promote insulin resistance, oxidative stress, and systemic inflammation, thereby contributing to the development of GDM. Conversely, metabolic disturbances accompanying GDM may further impair upper airway function and aggravate sleep-disordered breathing [9].

Although overnight polysomnography is considered the reference standard for diagnosing OSA, its routine use during pregnancy is limited by cost, availability, and patient burden. Consequently, questionnaire-based screening tools are frequently used to identify pregnant women who may be at increased risk for OSA. Among these, the Berlin Questionnaire and the STOP-BANG questionnaire are the most widely used instruments. The Berlin Questionnaire primarily evaluates snoring, daytime sleepiness, hypertension, and obesity, whereas the STOP-BANG questionnaire incorporates anthropometric characteristics together with classical OSA symptoms [10,11,12]. Because these instruments assess different domains of OSA risk, they may identify different groups of pregnant women as being at increased risk.

Previous studies investigating OSA risk during pregnancy have primarily focused on obesity, anthropometric characteristics, and pregnancy-related complications such as gestational hypertension and gestational diabetes mellitus. Although metabolic dysfunction and sleep-disordered breathing may share common mechanisms, limited evidence is available regarding the relationship between questionnaire-based OSA risk and diabetes-related family history. In addition, few studies have evaluated metabolic parameters and diabetes-related history using both the Berlin Questionnaire and STOP-BANG within the same pregnant population. Therefore, it remains unclear whether these screening instruments identify similar metabolic and familial risk patterns during pregnancy [13,14,15,16]. Studies conducted in Türkiye have previously examined questionnaire-based OSA risk in pregnant women and its relationships with anthropometric characteristics and adverse obstetric outcomes. However, evidence from Central Anatolia remains limited, and, to our knowledge, no previous regional study has concurrently compared the Berlin Questionnaire and STOP-BANG in relation to metabolic parameters and family history of diabetes. Evaluating these associations in a tertiary-care population in Central Anatolia may therefore provide locally relevant evidence and help address gaps in current antenatal screening strategies [17,18].

This study aimed to evaluate questionnaire-based OSA risk using the Berlin Questionnaire and the STOP-BANG questionnaire in pregnant women and to investigate the associations of metabolic parameters and diabetes-related history with questionnaire-based OSA risk. It was hypothesized that the Berlin Questionnaire would show stronger associations with adiposity, metabolic parameters, and diabetes-related family history than the STOP-BANG questionnaire in this pregnant population.

## 2. Materials and Methods

### 2.1. Study Design and Participants

This cross-sectional study was conducted between December 2025 and March 2026 at the obstetrics outpatient clinic of a tertiary care hospital in Central Anatolia, Türkiye. Ethical approval was obtained from the Yozgat Bozok University Social and Humanities Ethics Committee on 22 October 2025 (Approval No: 29/48). Written informed consent was obtained from all participants before enrollment, and the study was conducted in accordance with the Declaration of Helsinki. No a priori sample size calculation was performed. All eligible pregnant women who attended the outpatient clinic during the predefined study period and agreed to participate were consecutively included.

Pregnant women attending routine antenatal visits during the study period were screened for eligibility. Inclusion criteria were singleton pregnancy, age between 18 and 40 years, and willingness to participate. Women with a previous diagnosis of OSA, multiple pregnancy, chronic renal or hepatic disease, thyroid disease, psychiatric disorders, use of medications affecting sleep, or conditions preventing effective communication were excluded.

During the study period, 380 pregnant women attended the outpatient clinic. Of these, 260 met the eligibility criteria, and 210 agreed to participate and completed the study.

### 2.2. Data Collection

Data were collected through face-to-face interviews following routine obstetric examinations. Questionnaire responses were based on participant self-report and were not systematically verified with bed partners or household members. Sociodemographic and obstetric characteristics, including age, gestational week, educational status, employment status, smoking status, gravidity, parity, height, weight, family history of diabetes, and history of gestational diabetes mellitus (GDM), were recorded using a structured questionnaire developed from previous studies.

Anthropometric measurements were obtained during routine antenatal assessment. Body mass index (BMI) was calculated as weight (kg) divided by height squared (m^2^).

Biochemical parameters, including fasting plasma glucose, HbA1c, total cholesterol, HDL cholesterol, and LDL cholesterol, were obtained from fasting venous blood samples collected as part of routine antenatal care and extracted from the hospital electronic medical records.

### 2.3. Berlin Questionnaire

The Berlin Questionnaire is a validated screening instrument for identifying individuals at increased risk of OSA. It consists of ten questions grouped into three categories assessing snoring behavior, daytime sleepiness, and the presence of hypertension or obesity. Participants with positive scores in two or more categories were classified as being at high risk for OSA [9,11].

### 2.4. STOP-BANG Questionnaire

The STOP-BANG questionnaire consists of eight dichotomous (yes/no) items evaluating snoring, daytime tiredness, observed apnea, hypertension, BMI, age, neck circumference, and sex. Scores of 0–2 indicate low risk, 3–4 intermediate risk, and 5–8 high risk for OSA [10].

According to the STOP-BANG questionnaire, 183 participants were classified as low risk, 5 as moderate risk, and 22 as high risk. For the binary logistic regression analysis, the moderate- and high-risk categories were combined into a single elevated-risk group comprising 27 participants. This approach was adopted because the moderate-risk category included only five participants, and analyzing it separately could have resulted in sparse data and unstable multivariable estimates. The categories were combined only for the binary statistical analyses.

### 2.5. Statistical Analysis

Statistical analyses were performed using IBM SPSS Statistics version 25.0 (IBM Corp., Armonk, NY, USA). Continuous variables are presented as mean ± standard deviation (SD), whereas categorical variables are presented as frequencies and percentages.

The normality of continuous variables was evaluated using the Shapiro–Wilk test together with skewness and kurtosis values. Independent-samples *t*-tests were used to compare continuous variables between groups, while categorical variables were compared using the chi-square test or Yates’ continuity correction when appropriate.

Variables showing statistically significant associations in univariate analyses and those considered clinically relevant were entered into multivariable binary logistic regression models to identify factors independently associated with high questionnaire-based OSA risk according to the Berlin Questionnaire and the STOP-BANG questionnaire. HbA1c was analyzed as a continuous variable to preserve the full range of glycemic variation and to evaluate whether subtle differences in recent glycemic exposure were associated with questionnaire-based OSA risk. Odds ratios (ORs) with 95% confidence intervals (CIs) were calculated. Model fit was assessed using the Omnibus test, Hosmer–Lemeshow goodness-of-fit test, −2 log likelihood, Cox and Snell R^2^, Nagelkerke R^2^, and overall classification accuracy. A two-sided *p* value <0.05 was considered statistically significant.

## 3. Results

A total of 210 pregnant women were included in the study. According to the Berlin Questionnaire, 176 participants (83.8%) were classified as low risk and 34 participants (16.2%) as high risk for questionnaire-based OSA. According to the STOP-BANG questionnaire, 183 participants (87.1%) were classified as low risk, 5 participants (2.4%) as moderate risk, and 22 participants (10.5%) as high risk. For the binary logistic regression analysis, the 5 participants in the moderate-risk group and the 22 participants in the high-risk group were combined, resulting in a combined moderate/high-risk group of 27 participants. The distribution of questionnaire-based OSA risk is presented in Table 1.

The associations between sociodemographic and obstetric characteristics and questionnaire-based OSA risk are presented in Table 2. Gestational age, educational level, employment status, income level, smoking status, and history of gestational diabetes mellitus were not significantly associated with OSA risk according to either questionnaire (all *p* > 0.05). Older age was significantly associated with a higher questionnaire-based OSA risk according to both the Berlin Questionnaire (*p* = 0.024) and the STOP-BANG questionnaire (*p* = 0.003). Similarly, pregnant women with higher current body weight and BMI had significantly higher questionnaire-based OSA risk according to both questionnaires in the univariate analysis (all *p* < 0.05). However, BMI remained an independent predictor only in the multivariable logistic regression model based on the Berlin Questionnaire.

A family history of diabetes was significantly associated with high OSA risk according to the Berlin Questionnaire (*p* = 0.017), whereas no significant association was observed according to the STOP-BANG questionnaire (*p* = 0.720) (Table 2).

The relationships between biochemical parameters and questionnaire-based OSA risk are shown in Table 3. HbA1c, fasting plasma glucose, HDL cholesterol, total cholesterol, and LDL cholesterol were not significantly associated with OSA risk according to the Berlin Questionnaire (all *p* > 0.05). Likewise, no significant associations were observed between these biochemical parameters and OSA risk according to the STOP-BANG questionnaire (all *p* > 0.05) (Table 3).

Results of the multivariable logistic regression analysis for the Berlin Questionnaire are presented in Table 4. HbA1c, BMI, and a family history of diabetes were independently associated with high questionnaire-based OSA risk (all *p* < 0.05). Each one-unit increase in HbA1c was associated with a 1.446-fold increase in the odds of high questionnaire-based OSA risk, whereas each 1 kg/m^2^ increase in BMI increased the odds by 1.054-fold. In addition, participants with a family history of diabetes had approximately 2.8 times higher odds of being classified as high risk than those without a family history of diabetes. HDL cholesterol, LDL cholesterol, and a history of gestational diabetes mellitus were not independently associated with high questionnaire-based OSA risk.

The logistic regression model demonstrated acceptable calibration (Hosmer–Lemeshow *p* = 0.363), although its explanatory power was modest (Nagelkerke R^2^ = 0.137). The overall classification accuracy was 83.3%; however, only 2.9% of participants in the high-risk group were correctly classified (Table 4).

Results of the multivariable logistic regression analysis for the STOP-BANG questionnaire are presented in Table 5. None of the evaluated variables, including HbA1c, HDL cholesterol, LDL cholesterol, BMI, family history of diabetes, and history of gestational diabetes mellitus, was independently associated with high questionnaire-based OSA risk (all *p* > 0.05). The model showed acceptable calibration (Hosmer–Lemeshow *p* = 0.782), limited explanatory power (Nagelkerke R^2^ = 0.048), and an overall classification accuracy of 86.7% (Table 5).

## 4. Discussion

This study evaluated questionnaire-based OSA risk in pregnant women using the Berlin Questionnaire and the STOP-BANG questionnaire and examined its associations with metabolic parameters and diabetes-related history. Three main findings emerged. First, higher current body weight was consistently associated with increased questionnaire-based OSA risk according to both screening tools. BMI was associated with questionnaire-based OSA risk in the univariate analyses of both questionnaires but remained an independent predictor only in the multivariable model based on the Berlin Questionnaire. Second, a family history of diabetes was independently associated with high questionnaire-based OSA risk according to the Berlin Questionnaire but not according to the STOP-BANG questionnaire. Finally, none of the evaluated metabolic variables was independently associated with questionnaire-based OSA risk according to the STOP-BANG questionnaire.

Pregnancy is characterized by physiological changes that predispose women to sleep-disordered breathing, including weight gain, upper airway edema, and reduced functional residual capacity [12,13,14,15,16]. These changes become more pronounced as pregnancy progresses and may contribute to upper airway collapse during sleep. Consistent with previous studies, women with higher current body weight had greater questionnaire-based OSA risk in both screening instruments [19,20]. Although BMI was significantly associated with questionnaire-based OSA risk in the univariate analyses of both screening tools, it remained an independent predictor only in the multivariable logistic regression model based on the Berlin Questionnaire. This finding suggests that the association between BMI and questionnaire-based OSA risk identified by the STOP-BANG questionnaire may be influenced by other clinical variables included in the regression model. This finding is not unexpected because obesity represents an important component of the Berlin Questionnaire and has previously been shown to strongly influence its classification of OSA risk during pregnancy [20].

One of the most notable findings of the present study was the independent association between a family history of diabetes and high questionnaire-based OSA risk according to the Berlin Questionnaire. A family history of diabetes is associated with obesity, insulin resistance, and other cardiometabolic abnormalities that may increase susceptibility to sleep-disordered breathing [19,21]. However, this association was not observed with the STOP-BANG questionnaire. Because the Berlin Questionnaire and STOP-BANG evaluate different dimensions of OSA risk, they may classify pregnant women differently. Our findings therefore suggest that diabetes-related family history may be more closely associated with questionnaire-based OSA risk as assessed by the Berlin Questionnaire than by the STOP-BANG questionnaire. Nevertheless, because OSA was not confirmed by polysomnography, these findings should be interpreted as associations with questionnaire-based risk rather than with confirmed OSA. These variables should therefore not be regarded as established screening parameters for objectively diagnosed OSA. Rather, they represent instrument-specific associations with questionnaire-based risk that require confirmation using objective diagnostic methods.

In the multivariable logistic regression analysis, HbA1c was independently associated with high questionnaire-based OSA risk according to the Berlin Questionnaire, whereas fasting glucose, HDL cholesterol, LDL cholesterol, and a history of GDM were not. Although intermittent hypoxia has been linked to impaired glucose metabolism, marked glycemic disturbances may not yet have developed in a substantial proportion of participants, given the mean gestational age of the study population. The absence of significant associations with lipid parameters is also consistent with the heterogeneous findings reported in previous studies [19,22]. Although HbA1c was independently associated with Berlin Questionnaire-based risk, most values were within a non-diabetic range. Therefore, the magnitude of this association should not be assumed to represent a clinically meaningful glycemic difference or a diagnostic threshold. The finding should instead be regarded as exploratory and requires confirmation in larger cohorts with detailed metabolic assessment. A biologically plausible explanation for the observed association is that intermittent hypoxia and sleep fragmentation may increase sympathetic activation, oxidative stress, and systemic inflammation, thereby impairing insulin signaling and glucose regulation [8,19,23]. During pregnancy, these effects may interact with the physiological increase in insulin resistance and contribute to subtle variations in HbA1c, even among women without diagnosed diabetes [8,19,23]. Similarly, a family history of diabetes may reflect an underlying susceptibility to insulin resistance, obesity, and other cardiometabolic characteristics that overlap with risk factors for sleep-disordered breathing [24]. Nevertheless, these mechanisms were not directly evaluated in the present study, and the observed associations should not be interpreted as evidence of causality.

In contrast, no independent predictors of questionnaire-based OSA risk were identified using the STOP-BANG questionnaire. This may reflect differences in the structure of the two questionnaires as well as the characteristics of the study population. Importantly, the STOP-BANG questionnaire was originally developed for the general adult population and includes items such as male sex and age greater than 50 years, which are not applicable to pregnant women. These characteristics may reduce its discriminatory capacity in obstetric populations and should be considered when interpreting the findings. However, the absence of independent associations in the STOP-BANG model should be interpreted cautiously. The combined moderate/high-risk outcome included only 27 participants, and the null findings may therefore reflect limited statistical power and sparse outcome data rather than a true absence of association.

Pregnancy-specific prediction models and modified screening approaches have therefore been proposed, incorporating variables such as maternal age, pre-pregnancy BMI, chronic hypertension, and frequent snoring. Nevertheless, the available evidence suggests that the diagnostic performance of both conventional STOP-BANG and pregnancy-specific screening approaches remains variable. Accordingly, these tools may assist in identifying patients who require further assessment but should not replace objective diagnostic testing [25,26].

The regression model based on the Berlin Questionnaire demonstrated only modest explanatory power (Nagelkerke R^2^ = 0.137) and weak predictive performance. Although the model was statistically significant and showed acceptable calibration, it correctly classified only 2.9% of participants in the high-risk group. Therefore, the overall classification accuracy of 83.3% was largely driven by the correct classification of low-risk participants and should not be interpreted as evidence of adequate sensitivity. These findings substantially limit the model’s clinical usefulness for identifying individual pregnant women at elevated risk. Accordingly, the identified associations should be interpreted cautiously and regarded as exploratory associations with questionnaire-based OSA risk rather than as evidence of true OSA. Larger studies using objective diagnostic methods, particularly polysomnography, are needed to validate these findings.

## 5. Conclusions

In this cross-sectional study, the Berlin Questionnaire and the STOP-BANG questionnaire identified different patterns of questionnaire-based OSA risk among pregnant women. Higher current body weight and BMI were associated with increased questionnaire-based OSA risk in the univariate analyses of both screening tools. However, in the multivariable analysis, BMI, HbA1c, and a family history of diabetes remained independently associated with high questionnaire-based OSA risk only according to the Berlin Questionnaire. In contrast, HDL cholesterol, LDL cholesterol, and total cholesterol were not independently associated with questionnaire-based OSA risk in the multivariable models. Despite these statistically significant associations, the model’s modest explanatory power and extremely limited ability to identify high-risk participants preclude its use as a stand-alone clinical prediction model.

These findings reflect questionnaire-based risk classifications derived from self-reported screening instruments and should not be interpreted as evidence of objectively diagnosed OSA. Metabolic characteristics and diabetes-related history may influence questionnaire-based OSA risk differently depending on the screening instrument used. However, because OSA was not confirmed by polysomnography, these results should be interpreted with caution. Larger prospective studies incorporating objective diagnostic methods are needed to validate these findings and to clarify the role of metabolic factors in the assessment of OSA risk during pregnancy.

In routine antenatal care, questionnaire-based screening may serve as an initial risk-stratification approach, particularly among pregnant women with higher BMI or a family history of diabetes. However, these questionnaires should not be used as stand-alone diagnostic tools. Women classified as being at elevated questionnaire-based risk should undergo further clinical assessment and, when clinically indicated, objective sleep testing.

## 6. Limitations

This study has several limitations. First, OSA was assessed using the Berlin Questionnaire and the STOP-BANG questionnaire without polysomnographic confirmation, which remains the reference standard for the diagnosis of OSA. In addition, questionnaire responses regarding snoring, witnessed apnea, and daytime sleepiness were based on self-report and were not verified with bed partners, which may have resulted in underreporting or misclassification of symptoms. Therefore, the findings should be interpreted as associations with questionnaire-based OSA risk rather than confirmed OSA. Second, the STOP-BANG questionnaire was originally developed for the general adult population rather than pregnant women. Consequently, two of its items (male sex and age >50 years) were not applicable to our study population, which may have reduced its discriminatory performance. In addition, the moderate- and high-risk STOP-BANG categories were combined for binary statistical analyses because only five participants were classified as moderate risk. The relatively small number of participants in the combined moderate/high-risk group may have produced unstable estimates and limited the ability of the multivariable model to detect clinically relevant associations. Third, the study was conducted at a single tertiary care center with a relatively modest sample size, which may limit the generalizability of the findings. Furthermore, only 34 participants were classified as high risk according to the Berlin Questionnaire, while six variables were included in the multivariable model. This relatively low number of outcome events per variable may have increased the risk of overfitting and reduced the stability and generalizability of the regression estimates. Although neck circumference was considered within STOP-BANG scoring, continuous neck circumference measurements were not recorded as a separate variable and therefore could not be evaluated independently. Fourth, participants from different stages of pregnancy were analyzed together, and trimester-specific analyses were not performed. Although gestational week was not significantly associated with questionnaire-based OSA risk in the univariate analyses, dividing the sample into trimester-specific subgroups would have resulted in small numbers of moderate- or high-risk participants and potentially unstable estimates. Because physiological and metabolic characteristics vary throughout pregnancy, the absence of trimester-specific analyses may have influenced the observed associations. Finally, the explanatory power of the logistic regression model was modest, indicating that additional clinical and biological factors not evaluated in this study may contribute to questionnaire-based OSA risk. Future multicenter prospective studies incorporating polysomnography are warranted to validate these findings and clarify the role of metabolic factors in questionnaire-based OSA risk during pregnancy.

## Figures and Tables

**Table 1:** Distribution of OSA risk according to the Berlin and STOP-BANG questionnaires.

Questionnaires	Risk Level	n	%
**Berlin Questionnaire**	Low Risk	176	83.8
	High Risk	34	16.2
**STOP-BANG**	Low Risk	183	87.1
	Moderate Risk	5	2.4
	High Risk	22	10.5

**Table 2:** Comparison of selected sociodemographic and obstetric characteristics with Berlin and STOP-BANG questionnaire scores.

Continuous Variables	Berlin Low Risk (n = 176)		Berlin High Risk (n = 34)		Test	STOP-BANG Low Risk		STOP-BANG High Risk ^1^		Test/*p*
Age	27.75 ± 5.55		30.11 ± 5.55		2.270 * 0.024	27.69 ± 5.61		31.14 ± 4.67		−3.045 * 0.003
Gestational week	20.12 ± 14.12		21.55 ± 16.46		0.527 * 0.599	20.01 ± 14.75		22.70 ± 12.60		−0.900 * 0.369
Pre- pregnancy weight	62.84 ± 15.20		77.58 ± 19.36		4.195 * 0.001	64.42 ± 14.45		75.88 ± 18.99		−3.009 * 0.005
Current weight	67.08 ± 16.60		80.85 ± 21.92		4.188 * 0.001	67.39 ± 17.33		82.33 ± 19.18		−4.125 * 0.001
Current BMI	26.49 ± 4.73		31.25 ± 6.37		4.139 * 0.001	26.75 ± 4.77		30.70 ± 7.29		−2.727 * 0.011
	***n***	***%***	***n***	***%***		***n***	***%***	***n***	***%***	
Educational status										
Primary school	24	75.0	8	25.0	3.907 ** 0.272	27	84.4	5	15.6	2.410 ** 0.492
Middle school	36	80.0	9	20.0		38	84.4	7	15.6	
High school	57	85.1	10	14.9		57	85.1	10	14.9	
University and above	59	89.4	7	10.6		61	92.4	5	7.6	
Employment status										
Employed	28	96.6	1	3.4	4.026 *** 0.083	29	100.0	0	0	3.722 *** 0.054
Unemployed	148	81.8	33	18.2		154	85.1	27	14.9	
Income status										
Income less than expenses	23	88.5	3	11.5	1.410 ** 0.494	21	80.8	5	19.2	1.201 ** 0.549
Income equal to expenses	134	82.2	29	17.8		143	87.7	20	12.3	
Income greater than expenses	19	90.5	2	9.5		19	90.5	2	9.5	
Smoking status										
Yes	22	95.7	1	4.3	1.779 *** *0.182*	17	73.9	6	26.1	2.818 *** *0.093*
No	154	82.4	33	17.6		166	88.8	21	11.2	
Family history of diabetes										
Yes	32	71.1	13	28.9	5.667 *** ***0.017***	38	84.4	7	15.6	0.129 0.720
No	144	87.3	21	12.7		145	87.9	20	12.1	
History of GDM										
Present	5	83.3	1	16.7	0.001 *** 1.000	4	66.7	2	33.3	0.818 0.367
Absent	171	83.8	33	16.2		179	87.7	25	12.3	

* Independent sample *t*-test, ** Chi-square test, *** Yates’ correction applied.

^1^ The five moderate-risk participants were combined with the 22 high-risk participants only for binary statistical analyses.

**Table 3:** Comparison of selected biochemical parameters with Berlin and STOP-BANG questionnaire scores.

Variable	Berlin Low Risk	Berlin High Risk (n = 34)	Test	STOP-BANG Low Risk	STOP-BANG High Risk	Test/*p*
HbA1c	4.84 ± 1.50	5.32 ± 1.32	*1.172* *0.088*	4.77 ± 1.05	4.94 ± 1.54	*0.567* *0.571*
Fasting blood glucose	91.44 ± 67.55	93.64 ± 43.77	*0.182* *0.856*	92.43 ± 68.53	87.49 ± 16.06	*0.372* *0.710*
HDL	59.46 ± 25.01	61.78 ± 31.80	*0.472* *0.638*	59.22 ± 24.87	64.03 ± 33.91	*−0.891* *0.374*
LDL	96.84 ± 54.63	88.39 ± 49.55	*−0.837* *0.403*	95.06 ± 53.10	98.28 ± 59.50	*−0.554* *0.650*
Cholesterol	183.72 ± 66.54	191.56 ± 51.14	*0.650* *0.516*	181.84 ± 63.47	206.36 ± 66.71	*−1.862* *0.064*

**Table 4:** Binary logistic regression analysis of factors associated with high OSA risk according to the Berlin Questionnaire.

	B	S.E.	Wald	Sig.	Exp(B)	95% CI for Exp(B)	
						Lower	Upper
**Constant**	−4.797	1.347	12.684	0.001	0.008		
HbA1c	0.369	0.157	5.526	0.019	1.446	1.063	1.967
HDL	0.000	0.008	0.001	0.972	1.000	0.985	1.015
LDL	−0.005	0.005	1.185	0.276	0.995	0.986	1.004
BMI	0.053	0.022	5.741	0.017	1.054	1.01	1.102
Family history of diabetes	1.02	0.417	5.967	0.015	2.773	1.223	6.283
History of GDM	−0.434	1.18	0.135	0.713	0.648	0.064	6.545

Model fit statistics were as follows: Omnibus χ^2^ (6) = 17.634, *p* = 0.007; Hosmer–Lemeshow χ^2^ (8) = 8.758, *p* = 0.363; −2 Log Likelihood = 168.348; Cox & Snell R^2^ = 0.081; Nagelkerke R^2^ = 0.137; Overall classification accuracy = 83.3%.

**Table 5:** Binary logistic regression analysis of factors associated with high OSA risk according to the STOP-BANG Questionnaire.

	B	S.E.	Wald	Sig.	Exp(B)	95% CI for Exp(B)	
						Lower	Upper
**Constant**	−2.914	1.212	5.779	0.016	0.054		
HbA1c	−0.064	0.174	0.137	0.712	0.938	0.667	1.318
HDL	0.008	0.008	1.186	0.276	1.009	0.993	1.024
LDL	0.001	0.004	0.019	0.890	1.001	0.993	1.009
BMI	0.022	0.018	1.535	0.215	1.023	0.987	1.060
Family history of diabetes	0.258	0.487	0.281	0.596	1.294	0.498	3.360
History of GDM	1.150	0.908	1.604	0.205	3.158	0.533	18.717

Model fit statistics were as follows: Omnibus χ^2^ (6) = 5.477, *p* = 0.484; Hosmer–Lemeshow χ^2^ (8) = 4.764, *p* = 0.782; −2 Log Likelihood = 155.661; Cox & Snell R^2^ = 0.026; Nagelkerke R^2^ = 0.048; and an overall classification accuracy of 86.7%. Note: The five moderate-risk participants were combined with the 22 high-risk participants only for binary statistical analyses, resulting in a combined moderate/high-risk group of 27 participants.

## Data Availability

The datasets generated and analyzed in this study may be obtained from the corresponding author upon reasonable request, due to ethical constraints.
